# Intermittent catheterization in the management of post spinal cord injury (SCI) neurogenic bladder using new hydrophilic, with lubrication in close circuit devices – our own preliminary results

**Published:** 2012-03-05

**Authors:** A Spinu, G Onose, C Daia, C Panţu, A Anghelescu, L Onose, A Mihăescu

**Affiliations:** * The Teaching Emergency Hospital “Bagdasar-Arseni” (TEHBA), Bucharest, Romania; ** ”Carol Davila” University of Medicine and Pharmacy, Bucharest; *** Medical Express, Bucharest, Romania; **** The Medical Service of Metrorex, Bucharest, Romania

**Keywords:** intermittent catheterization, hydrophilic catheters, neurogenic bladder, spinal cord injury

## Abstract

This article is a review of the related approaches in the field – including the newest ones associated with a specific retrospective study on in-patients from our Clinic Division (preliminary results).

**Aim. Study design :** To objectively assess whether there are significant differences regarding some specific key biological and psychometric parameters related to the use of hydrophilic catheters vs. non-hydrophilic ones.

**Materials and Methods:** We have evaluated the outcomes of long term IC using by comparatively using the afore-mentioned two different types of catheters, on two lots (totally 45 patients with mainly retention type of neurogenic bladder): 30 post SCI patients, using exclusively hydrophilic catheters and respectively, 10 same kinds of patients that used exclusively non-hydrophilic catheters. Additionally, there were 5 patients included in both lots as they have started IC with non-hydrophilic catheters and since 2008 they have switched on using hydrophilic ones. The methods used were primary data acquisition based on a unitary questionnaire and statistical analyses.

**Results and discussion :** Mainly: the patients that used exclusively hydrophilic type of catheters (median: “None”) vs. those using exclusively non-hydrophilic type of catheters (median: “One every 4 months”) presented: a significantly lower number of inflammatory episodes at scrotal level (p-value: 0.0001 WT), a significantly lower number of post/intra/inter catheterization bleeding episodes (p-value: 0.0001 WT), a very slightly lower number of UTI activations and expressed a significant higher satisfaction level (p-value <0.0001 WT). However, speculating a conceptual relation with the lower number of inflammatory episodes at scrotal level, it is to be thought that bigger lots of patients could provide, in this respect, significant results too. This study is to be continued, in order to further validate these preliminary, quite promising results, on bigger lots through the complex/ rigorous assessment methodology already used.

**Abbreviations** IC – intermittent catheterism, SCI – spinal cord injury, CIC – clean intermittent catheterization, PVP – polyvinyl pyrolidone, UTI – urinary tract infections, WHO – World Health Organization, ICFDH – International Classification of Functioning, Disability and Health, PVC – polyvinyl chloride, RISCI - Initiation of a national informatics network for dynamic clustering of patients with spinal cord injury, QoL - Quality of life.

## Introduction

The neurogenic bladder is a severe illness that seriously impairs the quality of life (QoL) of patients, and at the same time, neurogenic bladder of retention type – prevailing met at SCI patients – may represent a real threat to their life.

The acute urinary retention is a condition resulted when the patient is unable to evacuate urine and may lead to bladder rupture through supradistension or/and vezico-ureteral reflux which leads – if the bladder is not emptied in time – to renal failure and even possibly exitus.

One of the most frequent causes of death in patients with spinal cord injury it used to be the renal failure subsequent to vezico-ureteral sub maximal, cvasipermanent outflow – but that was before the IC era [**1**]. So, the catheterization in an acute urinary retention, including due to a neurogenic bladder of retention type represents a major medical emergency.

A deficient management of avoiding – the absence of correct urinary catheterization regarding ritmicity, asepsis and antisepsis methods – represents a major cause of maintaining and aggravation of urinary tract infections (UTI), which may determine the impairment of the general state of the patient in the early post SCI periods and eventually the potential loss of renal function – with the prognostic above mentioned.

In order to decrease the complications rate and to increase the QoL of these individuals [**1**], a better understanding of the principles of neurogenic bladder management is required.

The other type of neurogenic bladder – the incontinent one – does not threaten directly the patients’ life, but seriously impairs its quality through the permanent discomfort determined by urine seeping on the underwear and even on skin, bearing to some very unpleasant situations, with a severe psychological trauma emphasized by his/her isolation in family and society, too.

Beside the psychosocial aspects, the urinary incontinence may also lead to skin lesions, which untreated, generate pressure sores.

### Functional classification of post SCI neurogenic bladder dysfunction

Traditionally there were only two classifications, but nowadays there are various related systems in order to facilitate and understand the management of this pathology.

From a brief historical perspective there are to be mentioned: the (first) functional one introduced by Lapides in 1970 (based on cystometric findings; it does not take into account the function of the sphincter mechanism), the first anatomical classification put in by Bors-Comarr (1971, considering the lesion level and the grade of completeness) and the next one, appeared in 1982 (Hald-Bradley – suprasacral/ infrasacral, being still in use; Madersbacher’s functional classification (1990, focused on therapeutic consequences: SCI level, detrusor and sphincter).

The actual complex functional classification in use is that of Krane-Siroky (1984), according to the passive bladder’s storage ability and the coordination of the detrusor/sphincter mechanism [**2**]. 

A combination of anatomic and functional classification is used in clinical practice, which we consider, to satisfactorily cover the complexity of this subject matter from a conceptual point of view: 

- the suprasacral post SCI neurogenic bladder which implies skeletal muscle spasticity, detrusor hyperreflexia, smooth sphincter synergia and striated sphincter dyssynergia and leads in a “fight” bladder retention type and (more rapid) renal failure or in incontinence.

- the sacral post SCI neurogenic bladder involves: detrusor areflexia, a non-relaxing but competent smooth sphincter and a low fixed tone striated sphincter, out of voluntary control and results more frequently in retention but with a much lower long term risk for hydronephrosis. This “flaccid” bladder kind permits IC under better circumstances [**3**].

The type of neurogenic bladder can be diagnosed using urodinamics, too.

Urodynamics may show the exact parameters of lower urinary tract function, pressure (40cmH2O is accepted as limit safe pressure during filling or voiding) and compliance of detrusor when the bladder is filling up and the maximal pressure of detrusor when discharging urine - which, together with the urinary debit, assess the manner of vezico-sphincterian function [**4,5**].

Although we meet in clinical practice also neurogenic bladder of pharmacological and psychological type, unfortunately this illness bases often on neural lesions (central or peripheral, specifically related to our study: post SCI) which explains its severity, especially that it is frequently irreversible.

It is also needful to mention that the matter of compensation the absence of mictional control is frequently for life time and not day by day, but many times a day and the users mainly start this harsh challenge around the age of 25 years old [**6**].

More, with a correct management (4 catheterizations/day and optimal conditions for their accomplishment) it is possible that their life not to differ significantly from the normal people, assuring in the same time a QoL rather close to normal, including by maintaining them in the professional side, so remaining contributors [**7**].

This way, we are touching, tangentially, the medico-psycho-socio-economic approach of the problem, because, being a medical demarche but day by day and all their life, inevitable it exceeds the clinical status, this theme closing a virtuous circle between medical component, high bio-technology, the prevention of an entire range of complications, QoL and the economic dimension: receiving these catheters for free.

This issue is being also an application of the ultramodern concept of human functioning elaborated of World Health Organization (WHO) in 2001 – International Classification of Functioning, Disabilities and Health (ICFDH) – which underlines the inseparable between bio/medical and social in human functioning [**8**].

### Intermittent Catheterization

Historical data

Catheterization is probably one of the oldest surgical procedures dating all the way back to 3000 years before Christ. The ancient Egyptians used to have catheters from bronze, but gradually they introduced those of gold, and even the wood ones. In the mid- 1930s, the American urologist F. Foley introduced the first balloon catheter – the Foley catheter [**9**].

In 1947, Guttman introduced the concept of sterile intermittent catheterization (natural desideratum from a principial point of view for every medical step, but difficult to apply especially in conditions of catheters technical performance in that époque).

In 1966, Guttman and Frankel presented the first long-term study about sterile intermittent catheterization [**10**].

Based on it and on an almost 7 decades of clinical practical use, intermittent catheterization (IC) has been validated and accepted as the state of the art in management of neurogenic bladder emptying disorders, in spinal cord injured people being life saving by reducing the risk of urosepsis and renal deterioration [**11**].

In 1972, Lapides introduced the concept of “clean intermittent catheterization” (CIC) an important contribution in IC, which meant a revolutionary improvement in the neurogenic bladder management. At the beginning IC was performed with polyvinyl chloride (PVC) catheters and later on, these ones were replaced by some low-friction catheters trying to reduce catheter-related complications (in Romania from 2008) [**12**].

At the same time, intermittent catheter, sterile water and collecting bag in one product offered the users more freedom and mobility and finally yet importantly achieved the goal for every invasive procedure: its sterility [**13**].

IC in management of neurogenic bladder (especially of retention type) individuals – including for SCI patients - was introduced as we previously showed up and it became the “gold standard” [**14**]. 

An extra argument for sustaining these issues is that analyzing (from the bacteriological point of view) the urine of Foley catheter’s users for IC, it was detected the infection with P. Mirabilis which may determine the encrustation and the constitute of crystalline biofilms [**15**].

And it is to be mentioned the relation between the bladder carcinoma and the persistent inflammation caused by the Foley catheter insertion [**16**].

The new technology has been improved, thus there is a tendency to “all in one” devices, with close circuit, sterile, pre-lubricated (sterile water and hydrophilic catheter in one product) – in Romania from 2010.

These catheters reduce urethral trauma and at the same time, improve autonomy of individuals.

Nowadays, IC is used most frequently in the management of neurogenic bladder patients also in traumatical brain injuries (TBI), multiple sclerosis, spina bifida/meningomielitis, bladder carcinoma, Parkinson disease, diabetus mellitus, post-partum/ surgery.

It is important to professional support patients on IC in order to obtain and further maintaining patients’ compliance [**6**].

The principle in IC is the cvasicomplete and regular voidance of the bladder (especially the sacrat bladder for which IC is achieving under better circumstances, improving social aspects of individuals) resulting a minimum post-mictional residuum and maintaining this way a low intravesical pressure associated with a decreased risk of vezico-ureteral outflow.

At the same time, this is the key for decreasing the risk of UTI including the secondary renal damage (leading on long term to renal failure). We have to mention that urethral catheters are the most prevalent cause of nosocomial UTI [**17**].

IC has also advantages on long-term use not only for bladder compliance and to infections rates but to bladder calculi and renal scarring, too [**18**].

The properties of catheter surface may influence an array of parameters: urethral complications, bacteriuria, UTI.

At the insertion and the removal of catheter, a low friction between its surface and the urethral mucous, it is extremely important in order not to damage it.

Consequently, when IC is used on a long-term period – as it happens, usually in neurogenic bladder of retention type – the specific characteristics of the catheter’s surface could minimize the risk for some late complications - frequent and significant: urethral strictures.

When polyvinyl pyrolidone (PVP) has been incorporated into the catheter surface, submersed in water it absorbs and binds it to the catheter, so the result is a slippery and smooth surface with a motion friction much reduced [**19**].

This new technology bases on a chemical process by which the catheter’s surface becomes isotonic, having the same salt concentration as urine. 

Here are some concrete examples of hydrophilic catheters with lubrication in close-circuit, being in clinical use (archive of The Neural-Muscular Rehabilitation Clinic Division of THEBA).

**Fig. 1:**
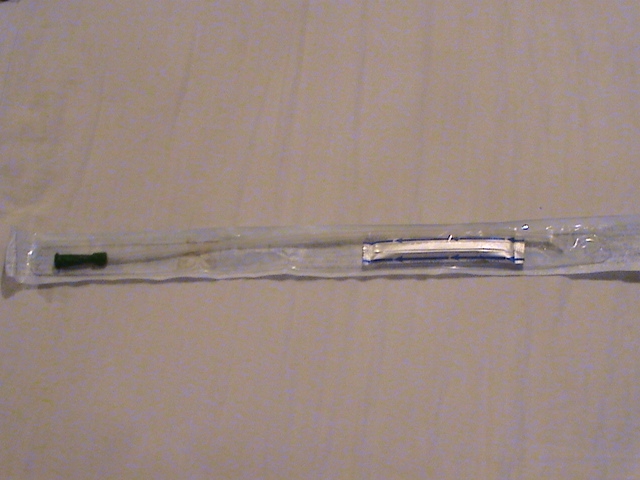
Hydrophilic catheter with lubrication in close-circuit without supplementary facilitations, this catheter being one of the two types found in Romania; their cost are fully supported by the National Assurance House for 4 catheters/day

**Fig. 2:**
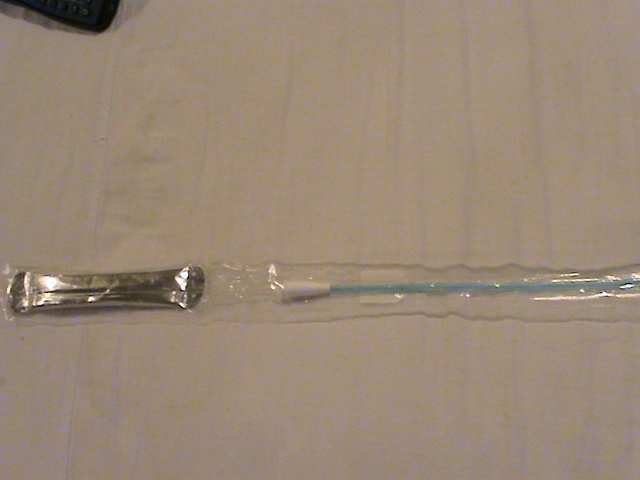
Hydrophilic catheter with lubrication in close-circuit without supplementary facilitations (different producer)

**Fig. 3:**
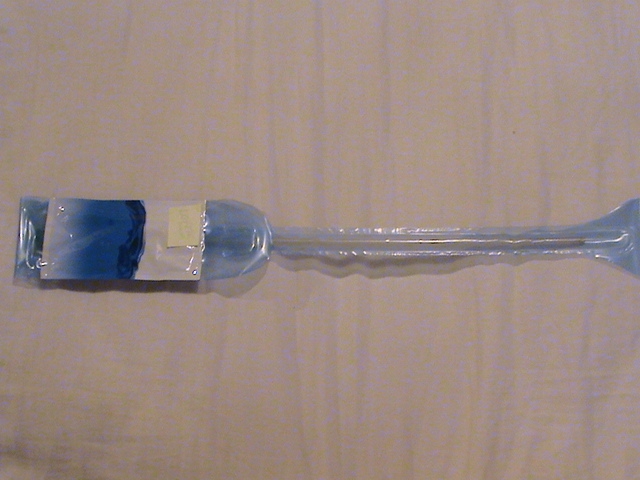
Hydrophilic catheter with lubrication in close-circuit with the facility of having a region impregnated with adhesive in order to fix it on an available surface (including a door or a wall) – very necessary, especially in self-catheterization

**Fig. 4:**
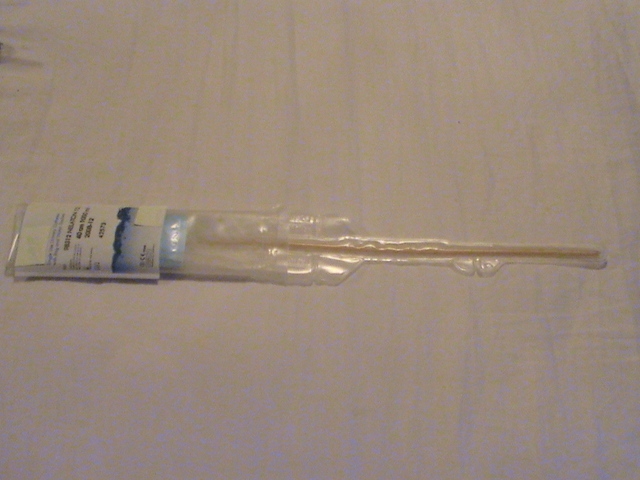
Hydrophilic catheter with lubrication in close-circuit: device with a collector bag incorporated

More than this, most of the patients using this novel type of catheter reported increased satisfaction (including general satisfaction), time spend, perception of IC and quality of life [**20**].

The compliance is better too, especially because of its acceptability and consequently the giving up rate in children and young adults is much smaller [**21**].

In Romania, subsequently to conjugate efforts including of the Physical and Rehabilitation (neural-muscular) Medicine – PR (n-m) M – Clinic Division of “Bagdasar- Arseni” Teaching Emergency Hospital in Bucharest since 2008 every patient with mainly retention type of neurogenic bladder may receive more sterile single use catheters/day undefined - based on some preliminary studies accomplished within the project with the acronym RISCI (Initiation of a national informatics network for dynamic clustering of patients with spinal cord injury, dedicated to improve their quality of life, by aiming the efficiency of the specific medico-social services, in transition), recently it has been adopted a public health programme which allow patients with neurogenic bladder to acquire 4 catheters/day, unlimited and for free [**22**].

Further, we present our preliminary clinical results related to the use of these new technological progresses.

In this context, we reiterate and underline the revolutionary progress represented by the passing from hydrophilic catheters with lubrication from exogen water (including distillate water) intake, to the lubrication in close circuit, assuring to the IC sterility comparable with the injectable procedures.

## Material and methods

We have comparatively evaluated the outcomes of long term IC using the afore mentioned two different types of catheters, on two lots (totally 45 patients): 30 post SCI patients with mainly retention type of neurogenic bladder, using exclusively hydrophilic catheters (M: 31; F: 4), aged between 19 (Min.) - 63 (Max.) years old (y.o.); Average (Av.): 43.85 y.o.; Median of age (M.o.a.): 45; Standard Deviation (S.d.): 13.60 and respectively, 10 same kind of patients that used exclusively non hydrophilic catheters (M: 13; F: 2) aged between 22 (Min.) - 62 (Max.) y.o.; Av.: 45.46 y.o.; M.o.a.: 47; S.d.: 13.10. To be noticed that 5 patients have been also their own witness, i.e. they are included in the both afore mentioned lots and therefore, we considered appropriate to re-group them, together, in a third, special lot: (M: 4; F:1) aged between 25 (Min.) - 57 (Max.) y.o.; A.v.: 42.8 y.o.; M.o.a.: 45; S.d.: 13.64.

Primary data acquisition was based on an unitary questionnaire (consisting of: name - hidden/ codified -, gender, age, oldness - mainly retention type - of neurogenic bladder, number of catheterizations/ day, time elapsed since the onset of IC, time elapsed since the onset of using hydrophilic catheterization, number of urinary tract infection - UTI – activations/ year, number of inflammatory episodes at scrotum level/ year, number of post/intra/inter catheterization bleeding episodes/ year; what type of catheters is preferable: Nelaton or Tieman?; patient’s subjective global appraisal on his/her level of satisfaction - on five degrees: much worse, worse, the same, better and much better - related to the IC procedure using hydrophilic catheters vs. non-hydrophilic ones) the answers being quantified as ordinal variables. Data assessment was based on the following methods: unvaried (using Somers’ concordance index - SI - and the Pearson correlation coefficient - PC), multivariate - using standardized canonical discriminator function coefficients - and respectively, the comparison Wilcoxon Two-Sample Test.

### Results and Discussion (preliminary):

Based on unvaried method, appeared a slight correlation between the satisfaction degree related to the IC procedure and the oldness of neurogenic bladder (SI: 0.2; PC: 0.219) and practically no concordance/ correlation between the first mentioned variable and: number of catheterizations/ day (SI: 0.078; PC: 0.039), time elapsed since the onset of IC (SI: 0.022; PC: 0.075), the number of urinary tract infection activations (SI: 0.035; PC: 0.084), inflammatory episodes at scrotal level (SI: 0.090; PC: 0,129), number of post/intra/inter catheterization bleeding episodes (SI: 0; PC: 0).

Completing the above mentioned method with a multivariate one (discrimination analysis applied on standardized data divided into two categories of patients’ answers – “Worse” and “Much worse”, respectively “Better” and “Much better” – related to their appraisal on the satisfaction level about the IC procedure) some of the above mentioned results have been elaborated, finally revealing the most contributive variables to this separation, are the time elapsed since the onset of IC (Standardized Canonical Discriminant Function Coefficients - SCDFC -: 2.238) and the time elapsed since the onset of using hydrophilic catheterization (SCDFC: 1.965); on the contrary, the oldness of neurogenic bladder seemed to have no influence over this separation (SCDFC: 0.001). 

**Fig. 5:**
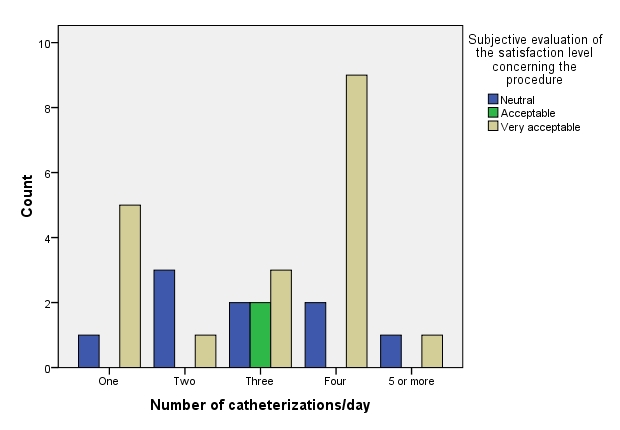
Number of catheterization/day

**Fig. 6:**
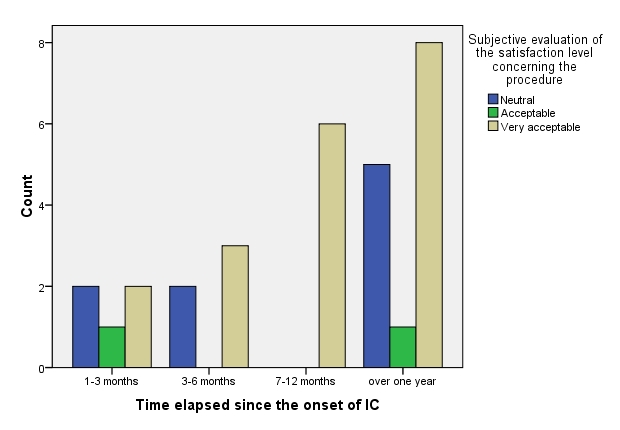
Time elapsed since the onset of IC

Regarding the comparative analysis (Wilcoxon Two-Sample Test -WT), it appeared that: 

- the (30) patients that used exclusively hydrophilic type of catheters (median: ”over 1 year”) vs. those (10) using exclusively non-hydrophilic ones (median: “7-12 months”) presented a slightly lower oldness of neurogenic bladder (p-value 0.026 WT;) 

**Fig. 7:**
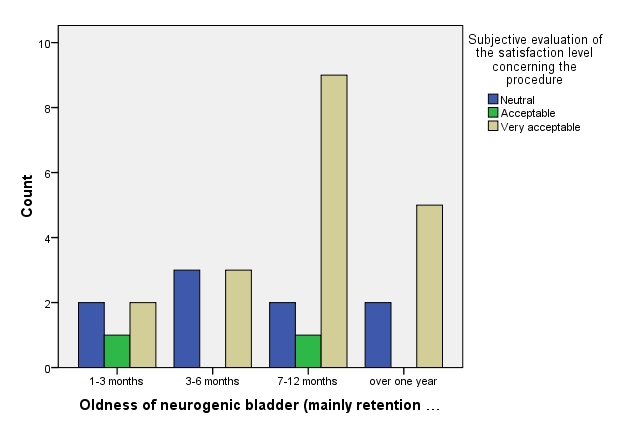
Oldness of neurogenic bladder (mainly retention type)

– the patients that used exclusively hydrophilic type of catheters (median: “None”) vs. those using exclusively non-hydrophilic type of catheters (median: “One every 4 months”) presented a significantly lower number of inflammatory episodes at scrotal level (p-value: 0.0001 WT)

**Fig. 8:**
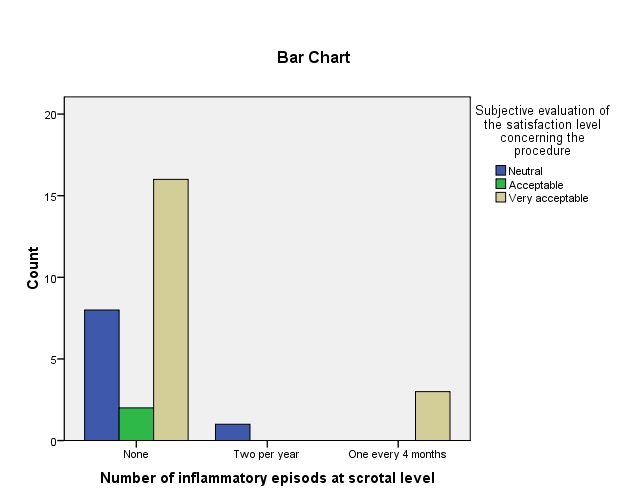
Number of inflammatory episodes at scrotal level

– the patients that used exclusively hydrophilic type of catheters (median: “None”) vs. those using exclusively non-hydrophilic type of catheters (median: “Two per year”) presented a significantly lower number of post/intra/inter catheterization bleeding episodes (p-value: 0.0001 WT).

**Fig. 9:**
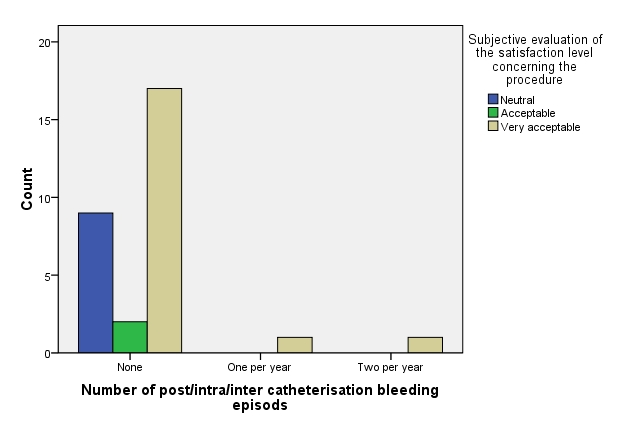
Number of post/inter catheterization-bleeding episodes

Although the special lot of 5 patients, who have been their own witnesses, is too small for statistical assessment, the observation of the primary data trend, regarding patients’ level of satisfaction towards the IC procedure (with hydrophilic vs. non-hydrophilic catheters) is resembling to the general one, resulting from the comparisons emphasized below:

– the patients that used exclusively hydrophilic type of catheters (median: ”Better”) vs. those using exclusively non-hydrophilic ones (median: ”Worse”) expressed a significant higher satisfaction level (p-value < 0.0001 WT )

**Fig. 10:**
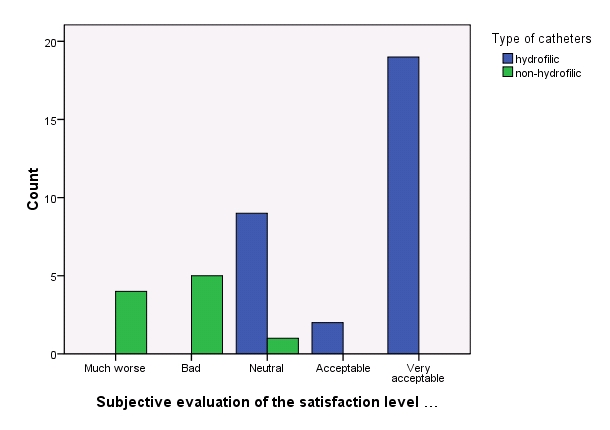
Subjective evaluation of the satisfaction level

Additionally, the patients that used exclusively hydrophilic type of catheters seem to prefer Tieman-type ones (median: “Much better”) vs. the Nelaton-type (median: “Better”; p=0.031), but statistically comparable.

**Fig. 11:**
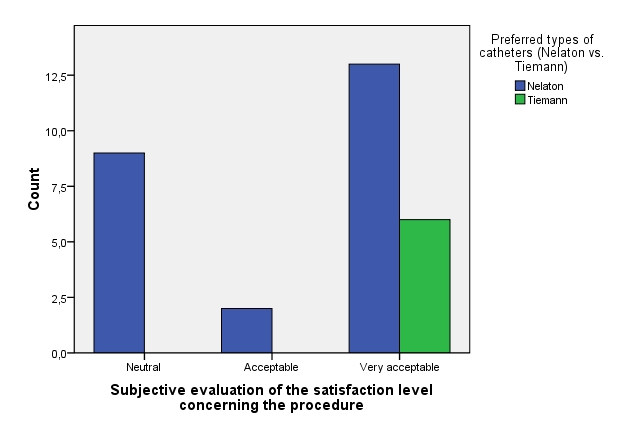
Subjective evaluation of the satisfaction level concerning procedure

At the same time, it resulted patients that used exclusively hydrophilic type of catheters (median: “Two per year”) vs. those using exclusively non-hydrophilic ones (median: “One every 4 months”) presented a very slightly lower number of UTI activations; therefore, it is very risky to accept the existence of a statistically significant related difference (p-value: 0.6274 WT).

**Fig. 12:**
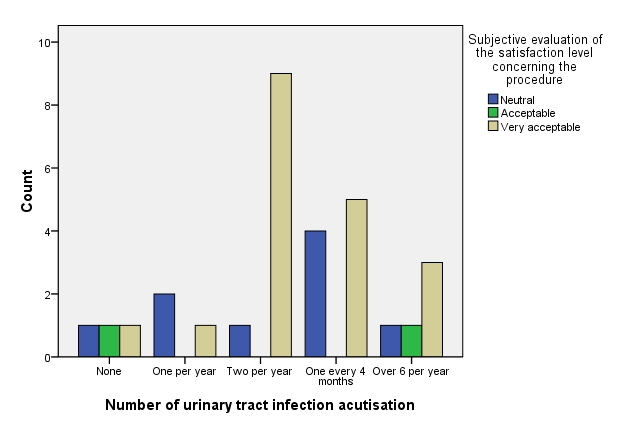
Number of urinary infection aggravation

However, speculating a conceptual relation with the lower number of inflammatory episodes at scrotal level, it is to be thought that larger lots of patients could provide, in this respect, significant results, too.

## Conclusion

Ensuring an IC with an appropriate rhythmicity and with hygienic-sanitary conditions (including minimal traumatization for urethra) to the neurogenic bladder patients represents an objective medical necessity and respectively an expression of European standards regarding the attitude of the sanitary system over a category of people profoundly misfortunate.

This study is to be continued, in order to further validate these preliminary, quite promising results on larger lots, through the complex/ rigorous assessment methodology already used.

## Competing Interests

We acknowledge the cooperation in the achievement of this paper with Medical Express, the first provider in Romania of hydrophilic catheters for IC in neurogenic bladder within a National Health Program
